# Improving the Standard of Care for All—A Practical Guide to Developing a Center of Excellence

**DOI:** 10.3390/healthcare9060777

**Published:** 2021-06-21

**Authors:** Elaina Vivian, Mary Rachel Brooks, Raquel Longoria, Laurie Lundberg, Jenifer Mallow, Jimmy Shah, Allison Vo, Alejandro Mejia, Paul Tarnasky, Vichin Puri

**Affiliations:** 1 Methodist Dallas Medical Center, Methodist Digestive Institute, 1441 N Beckley Ave., Dallas, TX 75203, USA; ElainaVivian@mhd.com (E.V.); MaryBrooks@mhd.com (M.R.B.); RaquelLongoria@mhd.com (R.L.); laurie.lundberg@att.net (L.L.); JeniferMallow@mhd.com (J.M.); JimmyShah@mhd.com (J.S.); AllisonVo@mhd.com (A.V.); PaulTarnasky@mhd.com (P.T.); 2 Methodist Dallas Medical Center, The Liver Institute, 1411 N Beckley Ave. #268, Dallas, TX 75203, USA; AlejandroMejia@mhd.com

**Keywords:** center of excellence, quality, pancreas, healthcare administration

## Abstract

Pancreatic surgery is one of the more challenging procedures performed by surgeons. The operations are technically complex and have historically been accompanied by a substantial risk for mortality and postoperative complications. Other pancreatic pathologies require advanced therapeutic procedures that are highly endoscopist-dependent, requiring specific, knowledge-based training for optimal outcomes. An increase in diagnosed pancreatic pathologies every year reinforces a critical need for experienced surgeons, gastroenterologists/endoscopists, hospitals, and support personnel in the management of complex pancreatic cases and thus, well-designed Centers of Excellence (CoE). In this paper, we outline the framework for a Pancreas CoE across three developmental domains: (1) establishing the foundation; (2) formalizing the program; (3) solidifying the CoE status. This framework can likely be translated to any disease or procedure-specific service-line and facilitate the development of a successful CoE.

## 1. Introduction

Pancreatic surgery remains one of the more challenging procedures performed by surgeons. The type of procedure depends on the location of the pathology [1]. From enucleation to total pancreatectomy, these operations are technically complex and have historically been accompanied by a substantial risk for mortality and postoperative complications [2,3]. Despite the complexity and morbidity associated with this type of surgery, the number of cases performed in the United States increased by 75% between 1993 and 2014 [4]. Additionally, there has been significant evolution in the surgical approaches available for pancreatic resections over the last eight decades. The first open pancreaticoduodenectomy was performed in 1935. The morbidity and mortality was more than 20% [5]. As technology and hospital systems improved, there was a decline in postoperative morbidity and mortality associated with pancreas surgery, which then allowed for other novel techniques to be applied to this discipline. It took almost 60 years before the first laparoscopic pancreaticoduodenectomy was performed in 1994 by Gagner and Pomp [6], but only another 7 years before the first robotic-assisted pancreaticoduodenectomy was performed by Giulianotti in 2001 [7]. To date, the majority of pancreatic resections are still performed using an open approach; however, minimally invasive and robotic approaches are on the rise due to certain advantages (e.g., reduced pain, complications, length of stay, blood loss, and bile leak rates) [8,9,10,11,12,13]. With an increase in diagnosed pancreatic pathology (symptomatic benign pancreatic cysts and pancreatic cancer), there will be a need for more surgeons that specialize in pancreas surgery. Given the advantages of minimally invasive and robotic techniques, surgeons must be trained in robotic surgical approaches through peer-to-peer instruction from experienced robotic surgeons in their specialty.

The incidence and severity of other pancreatic disorders, such as acute pancreatitis (AP), have also increased and are expected to continue to rise over time [14,15,16]. In the United States, AP is one of the leading gastrointestinal causes of hospitalization [16,17], with the most common cause being gallstone disease. Clinical practice guidelines recommend urgent endoscopic retrograde cholangiopancreatography (ERCP) within 24 h for patients with accompanied cholangitis [18,19,20]. ERCP happens to be one of the most advanced therapeutic procedures and has been found to be a highly endoscopist-dependent technique requiring specific, knowledge-based training in order to achieve technical competence [21]. Additionally, the utilization of endoscopic ultrasound techniques is critical for the diagnosis and treatment of a range of pancreatic and other indications, which also requires specific, knowledge-based training [22].

Research has shown a strong link between increased hospital and provider volume and improved patient outcomes [23,24,25,26]. In the Netherlands, less than desirable postoperative mortality rates prompted centralization of pancreatic surgery to regional high-volume hospitals [26]. After centralization, mortality rates decreased and two-year survival and overall survival after surgery increased compared to precentralization rates [26]. Lower endoscopist case volume was also found to be associated with higher failure rates for ERCP and greater need for postprocedure hospitalization [27,28,29,30]. This indicates a critical need for experienced surgeons, gastroenterologists/endoscopists, hospitals, and support personnel [31] in the management of complex pancreatic cases.

Together, the impact of centralized care on surgical outcomes in high-volume and specialized programs combined with the growing use of minimally invasive and robotic pancreatic surgical approaches has prompted the need for well-designed Centers of Excellence (CoE).

One hindrance of CoE development is the lack of an official definition of CoE. As a result, the rigor with which the term is used at one institution is often inconsistent with other institutions [32]. Although CoE connotes different structural levels at an institution, the term is often used to reference unique services aimed at treating a specific condition such as bariatric surgery, stroke care, or breast care. CoEs tend to provide a narrower range of services than a service line, but they offer high-level expertise with care delivered to patients in a comprehensive, multidisciplinary manner to achieve quality patient outcomes [33].

Healthcare administrators and physicians seek a framework that describes how to form a CoE and outlines what factors are essential to its success. However, current literature primarily focuses on only certain aspects of CoE development, such as the cost-savings potential or the impact on organizational dynamics [33]. This forces programs interested in establishing CoEs to have to start almost entirely from scratch, making them more susceptible to the time and cost consequences of trial and error.

Methodist Dallas Medical Center (MDMC) is an urban, tertiary referral teaching hospital located in Dallas, Texas. Methodist Dallas Medical Center established a well-defined, multidisciplinary Pancreas CoE as part of its branded “Methodist Digestive Institute” (MDI). Methodist Dallas Medical Center was designated the first Pancreas Surgery and Pancreatitis Disease-Specific Care (DSC) Certification program in the nation from The Joint Commission (TJC) as well as the first Pancreas Cancer DSC program in Texas.

In this paper, we outline the framework for a Pancreas CoE across three developmental domains:

• Establishing the foundation (i.e., leadership structure and purpose; financial considerations);
• Formalizing the program (i.e., clinical education and competency training; nurse navigation and multidisciplinary involvement; objective measures of clinical excellence; quality and performance improvement initiatives);
• Solidifying the CoE status (i.e., certification/accreditation by external institutions; marketing and outreach).

This framework can likely be translated to any disease- or procedure-specific service-line.

## 2. Establishing the Foundation

### 2.1. Leadership Structure and Purpose

**Figure 1:**
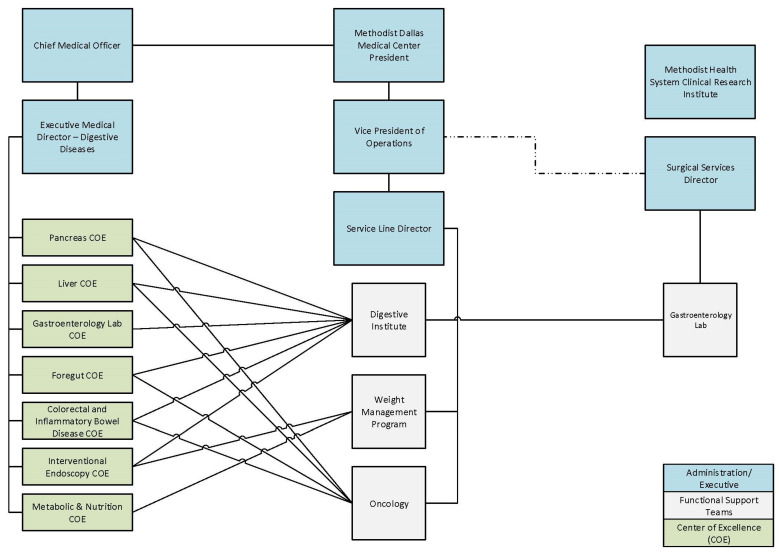
Matrix leadership structure.

**Figure 2:**
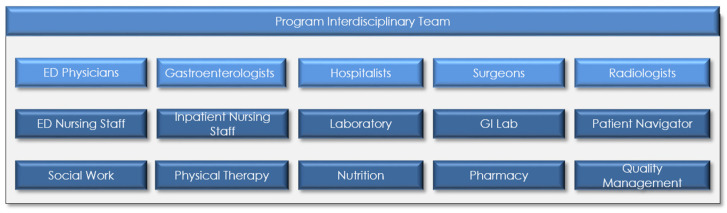
Interdisciplinary team of Pancreas CoE, ED = Emergency Department, GI = Gastroenterology.

A key priority in effective CoE development is establishing a leadership infrastructure capable of supporting the needs of patients and practitioners. This leadership infrastructure serves as the guiding element in a CoE, and can be relied upon to support evolving programmatic priorities. The MDI Pancreas CoE instituted a matrix leadership infrastructure that envelopes various specialty CoEs under the MDI umbrella (e.g., Pancreatic, Liver, or GI Lab) (Figure 1), and includes interdisciplinary team representation (Figure 2). It is important to note that pathologists and oncologists are also critically essential players on the interdisciplinary team, particularly for the management and treatment of patients with malignant pathologies.

Two essential leadership positions of the CoE are the medical director and the multidisciplinary program steering committee. The medical director fulfills several roles, including providing a voice for the program; assisting with physician alignment; establishing a brand for the program; serving as liaison between physicians, staff, and administrators; providing consistent feedback to administrators; assisting in gaining philanthropic support; and helping identify and generate performance initiatives. The multidisciplinary program steering committee has several responsibilities, including strategic and operational planning; clinical program development; instituting quality assurance/quality improvement initiatives; ensuring patient satisfaction; financial oversight (volume utilization, capital); education and fellowship support; and overseeing research and clinical trials.

### 2.2. Timeline of Pancreas CoE Development

The Pancreas CoE manages diseases related to pancreatic pathology, which includes benign and malignant pancreatic neoplasms and cysts, acute and chronic pancreatitis, and its sequela.

**Figure 3:**
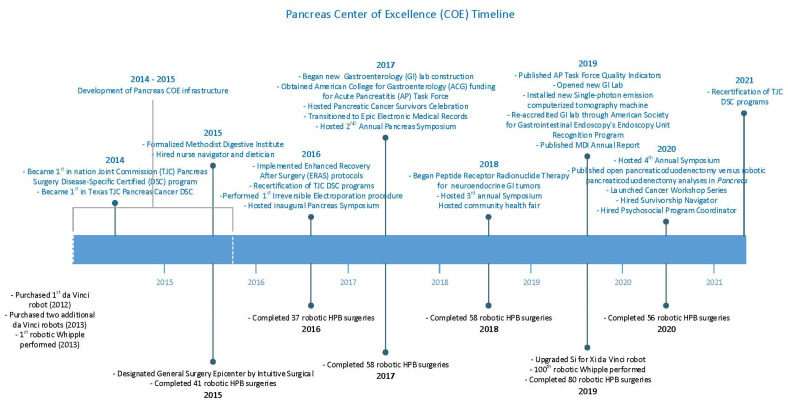
Pancreas CoE development timeline and major milestones.

The MDI began to build this Pancreas CoE in 2013 by establishing a program charter, which outlined what needed to be done and provided authorization to proceed and apply organizational resources. Figure 3 shows the Pancreas CoE development timeline and major milestones accomplished to date. The MDI Pancreas CoE program charter outlined the following objectives:

• Provide the highest standard of care, services, and support to each patient;
• Communicate process improvements and data to key stake holders in the pancreas domain;
• Analyze barriers and data to create better clinical pathways and care maps;
• Identify best practice guidelines and use them in our pancreas population;
• Identify quality and utilization metrics used to analyze physician practices.

The MDI Pancreas CoE also developed a formal mission statement to illustrate the program’s objectives and the approach used to reach those objectives: “To improve the care and outcomes of patients and families affected by pancreatic disease using a multidisciplinary team approach to deliver exceptional and compassionate care.” This statement aligns with MDI’s commitment to provide patients with education, research, and quality outcomes throughout the entire continuum of the care cycle.

### 2.3. Determining CoE’s Market Share

When developing a CoE, it is essential to identify a CoE’s market characteristics, including current market size and geographic location, future demand, and market share in current market(s). Administrators should conduct an analysis of the market characteristics to identify marketing opportunities and to set goals and priorities. Administrators should also conduct a competitive analysis to identify both facility and provider competitors. They should evaluate competitive advantages that can be leveraged, such as competing facilities and competing physicians.

### 2.4. Technology and Cost Considerations

Hospital administrators and providers must consider numerous costs when developing a CoE. Pro forma financial statements are typically developed in healthcare to formulate a prospectus of the possibilities before major investments are made towards new service lines or technology. A pro forma statement enables preinvestment analysis of cost and revenue impacts, providing insight into summary metrics such as the net present value, return on investment, and incremental rate of return [34].

A pro forma statement for a CoE should include details on training, staffing, certifications and accreditations, and minimally invasive technology. For example, the da Vinci Surgical System includes various cost components such as software, IT costs, construction, and the robot itself, which results in a need for an approximately $2.5 million investment. Following installation of a robotic surgical system, there are also annual IT upgrades, maintenance, and incidental costs that must be considered. Costs related to having surgeons and support staff trained on the surgical system of choice must also be factored in.

**Figure 4:**
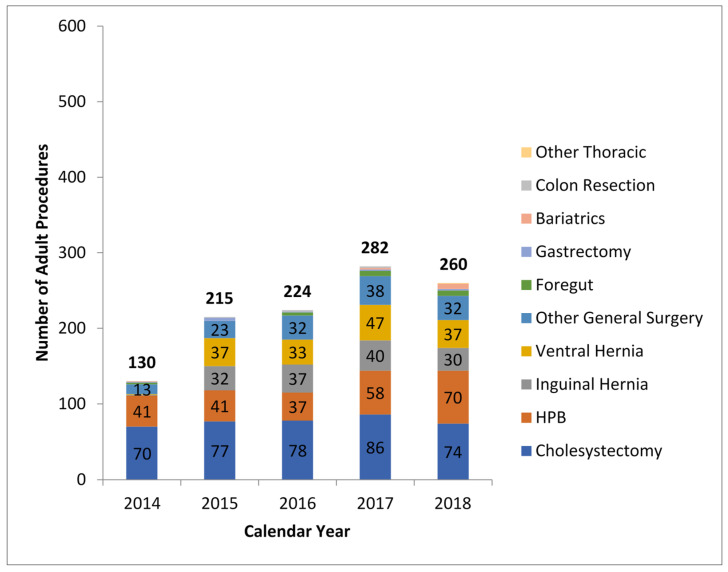
Yearly robotic procedure evolution.

The utilization of robotic technologies across service lines may enable fixed costs of robotic technology to be spread across a higher volume of cases, which can potentially make robotic surgery more cost-effective [35]. In the MDI experience, there has been a steady increase over the years in the number of HPBs and other surgeries utilizing the robotic approach (Figure 4). Leddy et al. discussed how the availability of inpatient beds can be limited by inpatient surgery volume and theorized that by decreasing length of stay, robotic surgery can potentially increase volume and further spread its fixed costs [36]. Additional robotic advantages, such as reduced pain, complications, and length of stay, may also result in decreased costs. Our own research demonstrated that hospital-stay charges were significantly lower for robotic pancreaticoduodenectomies, which supports the clinical finding that this group of patients went home faster and used significantly less hospital resources while in house, compared to those undergoing open pancreaticoduodenectomies [37].

Having advanced endoscopy technologies available in the GI suite is also a critical consideration for pancreatic CoEs. For example, the SpyGlass™ DS System is used not only for direct visualization of pancreatic and bile ducts, but also to evaluate suspected malignant and benign conditions, and for the treatment of difficult strictures and stones [38]. This technology has been shown to reduce the need for additional procedures [39] and subsequent costs [40] when used at the time of initial biliary stone treatments. Technology’s impact on quality and cost is an important consideration for value-driven CoEs and should be fully vetted during the development of the pro forma.

## 3. Formalizing the Program

### 3.1. Education/Competency/Training

Teaching and education are fundamental to a Pancreas CoE. Methodist Dallas Medical Center currently offers a gastrointestinal fellowship. Methodist Dallas Medical Center also has robust surgical and medical residency training programs managed by Methodist Health System’s Graduate Medical Education Department. All on-staff physicians at MDMC are required to be board certified in their area of specialty.

In addition to its fellowship programs, MDMC and MDI offers multidisciplinary physician education through the Liver Institute, a tumor conference in oncology, and a GI morbidity and mortality conference. Methodist Dallas Medical Center also offers external multidisciplinary learning opportunities (e.g., The Liver Institute’s Gut Club) for community physicians. Attendees at a monthly internal multidisciplinary journal club have an opportunity to share the latest clinical care practices, research publications, and health fairs targeted at high-risk populations in the community.

MDMC’s CoE offers a clinical lecture series, an annual symposium, and comprehensive educational courses that highlight advanced therapeutic techniques centered on live endoscopy cases treating a range of digestive diseases. High-definition teleconferencing and video services allow these real-time demonstrations to be directly transmitted from MDMC to off-site classrooms, facilitating peer-to-peer communication anywhere.

### 3.2. Multidisciplinary Involvement

A multidisciplinary environment is a distinguishing feature of a CoE. The collaborative team approach brings together practitioners from various disciplines, which improves quality of care by providing comprehensive treatment options [33]. Furthermore, work responsibilities and resources are divided within the institution to provide the best patient outcomes [33].

The nurse navigator is a crucial member of a pancreas multidisciplinary team. They coordinate, advocate for, educate, and support the patient and family throughout their journey at the CoE. The nurse navigator also facilitates access to resources and support networks that may be unfamiliar to patients and their families. Additionally, they provide evidence-based and familiar follow-up care. This level of expert guidance and compassionate care can give patients a sense of comfort [41], decrease patient anxiety during a high-stress hospitalization [42], improve patient satisfaction [43], and improve patients’ treatment compliance rates [44]. The navigator also assists with annual competency training for nursing staff along with providing learning opportunities to other members of the healthcare team by equipping them with educational materials, in-services, and performance measure tracking. The nurse navigator adds a vital service within a CoE by reducing barriers in care and streamlining a complex and continually evolving healthcare environment. Nurse navigators not only serve as a link between patients and providers within the multidisciplinary team, but they can also contribute to cost reduction and increased efficiency within an institution [45].

A registered dietitian is also an essential member of the pancreas multidisciplinary team. Dieticians work to identify the unique nutrition needs of each patient before and after surgery, support improved nutritional status, and provide individualized nutrition guidance and solutions. When originated preoperatively, nutrition management helps prepare each patient for the increased metabolic demands of surgery. Postoperatively, nutrition management helps support wound healing, promote functional recovery, and improve hospital length of stay [46].

### 3.3. Clinical Information Systems

Dashboards are powerful tools for a CoE. Dashboards simplify the process of data review by pulling together data from multiple sources into a visual format where the content is easily comprehensible. A dashboard provides physicians, nurses, and hospital administrators with relevant information about volume and outcomes so they can make informed decisions to improve operations and patient safety. Dashboards should include information that aligns with the needs of stakeholders (e.g., process, outcomes, and efficiency metrics; patient satisfaction; cost and resource utilization). They should also contain reliable and validated data, and visual elements such as run or control charts to identify significant trends [47]. When used correctly, dashboards serve to provide an objective assessment of hospital and/or physician performance. Implementing dashboards can improve adherence to clinical practice guidelines, change behaviors, and help improve patient outcomes [48]. Furthermore, comparing current performance to a benchmark can drive increased performance in motivated individuals [49].

MDMI developed group and individual physician dashboards for their Pancreas CoE program. The dashboards are stratified by disease and/or procedure to identify any patterns in the patient population. Dashboard data are reviewed quarterly at a pancreas multidisciplinary committee meeting to elicit feedback from physicians and other providers about performance. Data from the dashboard are pulled using a risk-adjusted tool that aggregates patient data from electronic health records. Risk adjustment ensures appropriate comparisons across unique patient populations by comparing inpatient cases to “like” cases by matching all patient-refined diagnosis-related groups (APR-DRG) and severity combinations. A risk-adjusted tool is available through Premier^®^ (https://www.premierinc.com, accessed on 17 June 2021). This system’s database contains data from hundreds of participating hospitals nationwide, allowing direct comparisons to the outcomes of other hospitals.

**Figure 5:**
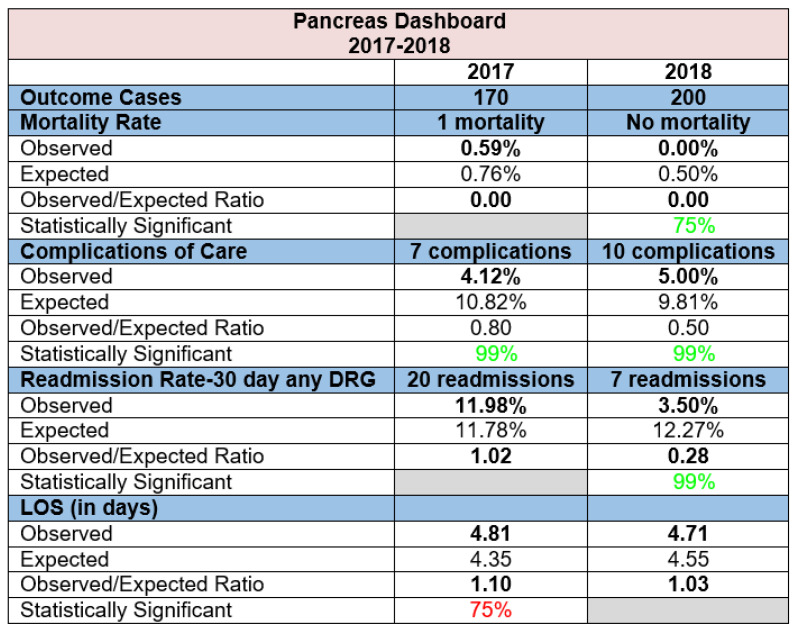
Example dashboard of clinical outcomes. Note: Green numbers represent the outcome’s variance is statistically better than expected; red numbers represent the outcome’s variance is statistically worse than expected; a grey box represents no statistical significance.

Dashboard metrics for MDMI’sCoE include volume, mortality rate, complications of care, 30-day readmissions, and length of stay (Figure 5). These metrics contain observed (a group or individual physician’s result) and expected (calculated by the risk adjustment methodology) values that are used to determine an observed/expected (O/E) ratio. Metrics with an O/E ratio less than 1.0 are performing better than expected, and outcomes with an O/E ratio greater than 1.0 are performing worse than expected. Variation is calculated by subtracting the expected value from the observed value. Outcomes with a negative variation are performing better than expected, and outcomes with a positive variation are performing worse than expected. Variation has three levels of statistical significance: 75%, 95%, and 99%. The level of detail included in the dashboard enables the multidisciplinary team to examine patient-level data and identify opportunities for improvement within the clinical practice.

### 3.4. Value-Based Healthcare

Many healthcare providers are adopting value-based healthcare models, as there has been a shift in the payment model from traditional fee-for-service to pay-for-performance bundle payments [50]. Value-based healthcare is defined as health outcomes achieved per dollar spent [51]. The goal of value-based healthcare is to encourage providers to improve overall patient care and experience while reducing costs. Value-based healthcare metrics typically cover several domains of patient care, such as clinical quality and safety (e.g., length of stay and readmission); processes of care (e.g., door-to-balloon time and surgery wait time); patient-reported outcomes (e.g., health-related quality of life), and patient feedback data (e.g., Hospital Consumer Assessment of Healthcare Providers and Systems surveys). The costs of the actual resources used to deliver outcomes over a full cycle of care, which are measured by patient and by condition, have been defined [52]. Costs can be broken down into categories (e.g., pharmacy, respiratory therapy, and room and board) and mapped to determine trends or significant variation, which enables improvement initiatives on problematic resource use issues.

In 2019, MDMI’s CoE initiated perfect care index tracking. The team selected significant clinical and patient metrics for two patient populations: acute pancreatitis (AP) patients and pancreatic cancer patients who undergo a Whipple procedure. If data abstracted from a patient’s electronic health record showed the patient met or exceeded the desired outcomes for each metric, the patient was considered to have received “perfect care” [53]. Together, the use of perfect care index tracking (the percentage of the total number of patients who met perfect care) and cost data (the average cost of patient care split into various cost categories) can constitute a visual representation of value-based healthcare [52].

In 2015, MDMC implemented standardized order sets for managing patients with AP in order to achieve treatment goals, called the Methodist Acute Pancreatitis Protocol (MAPP). The MAPP order sets were based on clinical practice guidelines [19], which were applied in the emergency department after initial diagnosis of AP and again upon the patient’s initial admission. Physicians could provide additional orders as deemed appropriate throughout the patients’ hospital stay. Perfect care metrics for AP included in-hospital mortality, 30-day readmission, LOS less than or equal to that expected based on risk-adjusted data, computed tomography (CT) scan ordered in the ED, Lactated Ringers (LR) administered in ED, ERCP performed within 24 h of diagnosis for patients with cholangitis, blood urea nitrogen (BUN) and hematocrit (used as surrogates for hydration) on the day patient presents to the ED and the following day, total parenteral nutrition (TPN) usage, oral nutrition within 72 h, and final severity of AP. Patients had to meet all goals in order to achieve perfect care (Table 1). For other pancreatic indications or associated procedures, such as emergent endoscopy, CT-guided drainage, or interventional radiologic procedures, evidence-based perfect care metrics would need to be established and monitored separately.

MDMI found that in 2013–2014, prior to MAPP being implemented, 5.3% of patients met criteria for perfect care and had average treatment costs of $41,755. In 2018–2019, 4 years after MAPP implementation, 35.3% of patients met criteria for perfect care, a sizable increase from 2013–2014 (*p* < 0.0001), with no significant increase in average inflation-adjusted total charges (*p* = 0.8262) [54].

### 3.5. Quality/Performance Improvement Outcomes

CoEs need a structure in place to act on the opportunities for improvement identified from the clinical outcomes dashboards and perfect care index tracking. Our Pancreas CoE established Performance Improvement Workgroups that are composed of multidisciplinary providers and other staff members actively involved in patient care. The goal of these workgroups is to develop performance improvement interventions, either proactively or in response to identified issues. These interventions are constructed as SMART (Specific, Measurable, Achievable, Relevant, and Time-bound) goals and incorporate statistical process control applications (e.g., run/control charts) and other quality improvement tools like fishbone diagrams, root cause analyses, and Plan-Do-Study-Act cycles.

The American College of Gastroenterology (ACG) published guidelines regarding the management of AP in 2013 [19], which recommended abdominal ultrasound for initial imaging. The guidelines stated that contrast-enhanced CT and/or MRI should be reserved only for patients with an unclear diagnosis or for those who fail to improve clinically within the first 48 to 72 h. Data from AP patients managed prior to the 2013 guidelines suggested a need to improve initial imaging use [55]. MDMI’s CoE provided education to encourage physicians to use the MAPP order sets to manage patients with suspected or confirmed AP. After the introduction of MAPP, MDMI’s CoE achieved a 50.4% reduction in the use of CT scans on presentation without an associated increase in final severity outcome [55].

## 4. Solidifying the CoE Status

### 4.1. Certifications and Designations

TJC certification provides numerous benefits that can set a CoE apart as a quality destination for pancreas surgery. Certification improves quality of care by decreasing variation in clinical processes, subsequently reducing risk of error [56]. In addition, certification provides an objective assessment of clinical performance. TJC surveyors offer expert advice and education on best practices during intracycle and on-site reviews [56]. Having a TJC Gold Seal of Approval^®^ will likely increase community and provider awareness and confidence in the CoE as a destination for quality pancreas care, leading to increased patient volume [56]. TJC provides a list of standards that encompass all the necessary criteria to earn and maintain a certification award [57]. TJC assesses compliance with these standards during on-site reviews. Those standards help the organization develop an environment that encourages continuous improvement in patient care.

Similarly, DNV-GL Healthcare is another organization that provides certifications to acute care and critical access hospitals for “meeting or exceeding standards of clinical readiness and patient safety” [58].

Other organizations such as the makers of the da Vinci Surgical System, Intuitive Surgical, also provide designations to hospitals and surgeons who have demonstrated expertise in robotic-assisted surgical procedures that are teachable, reproducible, and effective. MDMI’s CoE and affiliated, independently practicing surgeons exemplify such expertise, specifically in complex pancreas and HPB surgical procedures, and were designated in 2015 as an Epicenter for General Surgery by Intuitive Surgical. Surgeons from around the world have traveled to Dallas to observe and learn specialized HPB robotic-assisted surgical techniques using the da Vinci Surgical System. The designation for general surgery means that MDMC is one of only five centers in Texas and the first to be focused on HPB robotic-assisted surgery [59].

### 4.2. Marketing a CoE

Although having a CoE helps to establish the reputation and credibility of the program or service, much must be done to ensure the service becomes known as a referral center of choice within the local community, region, and nation. A thorough analysis will help uncover the distinct attributes of the services, providers, and organization as well as the unique opportunities that will enable an organization to introduce and maintain a successful market presence. The analysis includes evaluating market research and outreach such as billboards; physician office visits; publication of academic, peer-reviewed manuscripts and white papers; community education (health fairs, online publications (e.g., https://researchoutreach.org/articles/robotics-the-future-of-liver-surgery/, accessed on 21 June 2021), support groups, etc.), and continuing medical education events.

Additional marketing considerations include the following:

• The PLC is a beneficial tool that helps marketers manage the stages of a product’s acceptance and success in the marketplace. The PLC begins with the product’s introduction and continues through its growth in market share, maturity, and possible decline in market share [60]. Where the CoE lands in the program life cycle (e.g., introductory, growth, maturity, or decline) must be evaluated to help determine appropriate messaging.
• Legal and ethical issues: Prior to embarking on CoE marketing, it is important to consider the legal and ethical ramifications of medical marketing. Consider the following questions with an organization’s legal and marketing teams:
  ○ Are all quality claims backed by evidence-based criteria? Objective claims regarding experience, competence, and the quality of physicians and the services they provide may only be made if they are factually supportable [61]. In addition, be mindful that marketing materials, including websites, should be reviewed and updated regularly (at least annually, or as changes occur) to ensure that claims continue to be accurate.
  ○ What are the organization’s policies regarding advertising individual providers?
  ○ Are there disclaimers that must be used in marketing materials?
  ○ Do brand guidelines dictate the identity of services?
• Provider expertise: Several factors lend to the reputation of an organizations’ providers, and subsequently the program. One factor is being a teaching facility, such as the Intuitive Surgical Training Epicenters, or having surgeons who are board-certified and fellowship-trained. Another factor is identifying physician leaders, champions, and subject matter experts among specialists to determine those most able and willing to help develop communications materials and participate in marketing efforts.
• Defining the market and audience: Identify the target audience before planning a promotional mix. Both providers (e.g., physicians, physician assistants, and nurse practitioners) and direct consumers comprise the target audience for services. Messaging should be crafted to meet the needs and level of understanding of the target audience.
• Setting marketing goals: Establish baselines for marketing metrics and develop SMART goals for each. Goals might include increasing procedure volume, increasing referrals, expanding referral base, increase market share, and achieving return on investment from marketing expenditures.
• Budgeting and tracking results: Before marketing the CoE, know the organization’s budgeting cycle. The budget will dictate the promotional mix. A marketing consultant can provide cost estimates for the tools and tactics they recommend. Results should be tracked against the original baseline SMART goals and metrics established at the onset of planning efforts.

## 5. Conclusions

In order to achieve a Pancreas CoE, MDI proposes that first, there needs to be a consistent definition of CoE among all institutions. Second, all HPB surgeons should be trained in robotic surgical approaches through peer-to-peer instruction and multidisciplinary staff be trained in their area of expertise. Third, MDI proposes three essential developmental domains that will help in improving organizational dynamics: establishing the foundation, formalizing the program, and solidifying the CoE status.

The first domain, establishing the foundation, ensures that leadership is able to support patients and staff. For example, MDMC provided necessary resources, a mission statement, marketing analysis, and a pro forma statement in order to begin to build its CoE. For the second domain, formalizing the program, MDMC provided extensive clinical education and training, and multidisciplinary involvement such as a nurse navigator and dietician. For the final domain, solidifying the CoE, MDMC obtained certification and accreditation from The Joint Commission (TJC) and was designated as an Epicenter General Surgery by Intuitive Surgical, which allows for numerous benefits, such as labeling MDMC as a quality destination for minimally invasive and robotic HPB surgeries. Following the steps outlined in this paper can support other facilities’ work towards building a Pancreas or other disease- or procedure-specific CoE. However, a potential limitation of this framework includes acknowledgment that some of the economic aspects outlined are valid in the United States but may not be immediately transferable or relevant to facilities in other countries. All facilities interested in building a Pancreas or other disease- or procedure-specific CoE should adapt this framework according to their systemic needs.

## Figures and Tables

**Table 1:** Perfect care index metrics and goals.

Perfect Care Index Metric	Goal
**Clinical Quality and Safety**	
In-hospital mortality	No
30-day readmission	No
Length of stay	≤Expected based on risk-adjusted data
Final severity of pancreatitis	Mild or moderate severity
**Process of Care**	
Computed tomography scan ordered in the Emergency Department	No
Lactated ringer’s administered in the Emergency Department	Yes
ERCP ^†^ performed within 24 h for patients with cholangitis	Yes
BUN **^‡^** and HCT **^§^**	Decrease from day 0 to day 1
Total parenteral nutrition usage	No
Oral nutrition	Within 72 h

Note: ^†^ ERCP = Endoscopic Retrograde Cholangiopancreatography; ^‡^ BUN = Blood Urea Nitrogen (BUN); ^§^ HCT = Hematocrit (used as surrogates for hydration).

## Data Availability

Data sharing not applicable: No new data were created or analyzed in this study. Data sharing is not applicable to this article.

## References

[1] Nassour I., Choti M.A. (2016). Pancreatic Operations. JAMA.

[2] Zovak M., Mužina Mišić D., Glavčić G. (2014). Pancreatic surgery: Evolution and current tailored approach. Hepatobiliary Surg. Nutr..

[3] Nimptsch U., Krautz C., Weber G.F., Mansky T., Grutzmann R. (2016). Nationwide In-hospital Mortality Following Pancreatic Surgery in Germany is Higher than Anticipated. Ann. Surg..

[4] Agency for Healthcare Research and Quality (2019). Healthcare Cost and Utilization Project (HCUP).

[5] Tan-Tam C., Segedi M., Chung S. (2016). Whipple procedure: Patient selection and special considerations. Open Access Surg..

[6] Gagner M., Pomp A. (1994). Laparoscopic pylorus-preserving pancreatoduodenectomy. Surg. Endosc..

[7] Giulianotti P.C., Coratti A., Angelini M., Sbrana F., Cecconi S., Balestracci T., Caravaglios G. (2003). Robotics in general surgery: Personal experience in a large community hospital. Arch. Surg..

[8] Thornblade L.W., Shi X., Ruiz A., Flum D.R., Park J.O. (2017). Comparative Effectiveness of Minimally Invasive Surgery and Conventional Approaches for Major or Challenging Hepatectomy. J. Am. Coll. Surg..

[9] Nguyen K.T., Gamblin T.C., Geller D.A. (2009). World review of laparoscopic liver resection-2804 patients. Ann. Urgery.

[10] Schiffman S.C., Kim K.H., Tsung A., Marsh J.W., Geller D.A. (2015). Laparoscopic versus open liver resection for metastatic colorectal cancer: A metaanalysis of 610 patients. Surgery.

[11] Sham J.G., Richards M.K., Seo Y.D., Pillarisetty V.G., Yeung R.S., Park J.O. (2016). Efficacy and cost of robotic hepatectomy: Is the robot cost-prohibitive?. J. Robot. Surg..

[12] Bagante F., Spolverato G., Strasberg S.M., Gani F., Thompson V., Hall B.L., Bentrem D.J., Pitt H.A., Pawlik T.M. (2016). Minimally Invasive vs. Open Hepatectomy: A Comparative Analysis of the National Surgical Quality Improvement Program Database. J. Gastrointest. Surg..

[13] Rao A., Rao G., Ahmed I. (2012). Laparoscopic vs. open liver resection for maignant liver disease. A systematic review. Surgeon.

[14] Yadav D., Lowenfels A.B. (2013). The epidemiology of pancreatitis and pancreatic cancer. Gastroenterology.

[15] Munigala S., Yadav D. (2016). Case-fatality from acute pancreatitis is decreasing but its population mortality shows little change. Pancreatology.

[16] Peery A.F., Crockett S.D., Murphy C.C., Lund J.L., Dellon E.S., Williams J.L., Jensen E.T., Shaheen N.J., Barritt A.S., Lieber S.R. (2019). Burden and Cost of Gastrointestinal, Liver, and Pancreatic Diseases in the United States: Update 2018. Gastroenterology.

[17] Peery A.F., Dellon E.S., Lund J., Crockett S.D., McGowan C.E., Bulsiewicz W.J., Gangarosa L.M., Thiny M.T., Stizenberg K., Morgan D.R. (2012). Burden of gastrointestinal disease in the United States: 2012 update. Gastroenterology.

[18] Lee H.S., Chung M.J., Park J.Y., Bang S., Park S.W., Song S.Y., Chung J.B. (2018). Urgent endoscopic retrograde cholangiopancreatography is not superior to early ERCP in acute biliary pancreatitis with biliary obstruction without cholangitis. PLoS ONE.

[19] Tenner S., Baillie J., DeWitt J., Vege S.S. (2013). American College of Gastroenterology guideline: Management of acute pancreatitis. Am. J. Gastroenterol..

[20] Vivian E., Cler L., Conwell D., Cote G.A., Dickerman R., Freeman M., Gardner T.B., Hawes R.H., Kedia P., Krishnamoorthi R. (2019). Acute Pancreatitis Task Force on Quality: Development of Quality Indicators for Acute Pancreatitis Management. Am. J. Gastroenterol..

[21] Jovanovic I., Mönkemüller K. (2018). Quality in endoscopy training-the endoscopic retrograde cholangiopancreatography case. Ann. Transl. Med..

[22] Cho C.M., Dewitt J., Al-Haddad M. (2011). Echo-endoscopy: New therapeutic frontiers. Minerva Gastroenterol. Dietol..

[23] Lieberman M.D., Kilburn H., Lindsey M., Brennan M.F. (1995). Relation of perioperative deaths to hospital volume among patients undergoing pancreatic resection for malignancy. Ann. Surg..

[24] Orr R.K. (2010). Outcomes in pancreatic cancer surgery. Surg. Clin. N. Am..

[25] Adam M.A., Thomas S., Youngwirth L., Pappas T., Roman S.A., Sosa J.A. (2017). Defining a Hospital Volume Threshold for Minimally Invasive Pancreaticoduodenectomy in the United States. JAMA Surg..

[26] Lemmens V.E.P.P., Bosscha K., van der Schelling G., Brenninkmeijer S., Coebergh J.W.W., de Hingh I.H.J.T. (2011). Improving outcome for patients with pancreatic cancer through centralization. BJS.

[27] Coté G.A., Imler T.D., Xu H., Teal E., French D.D., Imperiale T.F., Rosenman M.B., Wilson J., Hui S.L., Sherman S. (2013). Lower provider volume is associated with higher failure rates for endoscopic retrograde cholangiopancreatography. Med. Care.

[28] Keswani R.N., Qumseya B.J., O’Dwyer L.C., Wani S. (2017). Association Between Endoscopist and Center Endoscopic Retrograde Cholangiopancreatography Volume With Procedure Success and Adverse Outcomes: A Systematic Review and Meta-analysis. Clin. Gastroenterol. Hepatol..

[29] Huang R.J., Barakat M.T., Girotra M., Lee J.S., Banerjee S. (2019). Unplanned Hospital Encounters After Endoscopic Retrograde Cholangiopancreatography in 3 Large North American States. Gastroenterology.

[30] Lee H.J., Cho C.M., Heo J., Jung M.K., Kim T.N., Kim K.H., Kim H., Cho K.B., Kim H.G., Han J. (2020). Impact of Hospital Volume and the Experience of Endoscopist on Adverse Events Related to Endoscopic Retrograde Cholangiopancreatography: A Prospective Observational Study. Gut Liver.

[31] D’Angelica M.I., Chapman W.C. (2016). HPB Surgery: The Specialty is Here to Stay, but the Training is in Evolution. Ann. Surg. Oncol..

[32] Why Is a ‘Center of Excellence’ Different from an Institute?. https://www.advisory.com/research/market-innovation-center/the-growth-channel/09/what-is-the-difference-between-a-center-of-excellence-and-an-institute.

[33] Elrod J.K., Fortenberry J.L. (2017). Centers of excellence in healthcare institutions: What they are and how to assemble them. BMC Health Serv. Res..

[34] Bata S.A., Richardson T. (2018). Value of Investment as a Key Driver for Prioritization and Implementation of Healthcare Software. Perspect. Health Inf. Manag..

[35] Perez R.E., Schwaitzberg S.D. (2019). Robotic surgery: Finding value in 2019 and beyond. Ann. Laparosc. Endosc. Surg..

[36] Leddy L.S., Lendvay T.S., Satava R.M. (2010). Robotic surgery: Applications and cost effectiveness. Open Access Surg..

[37] Mejia A., Shah J., Vivian E., Acharya P. (2020). Analysis of 102 Fully Robotic Pancreaticoduodenectomies: Clinical and Financial Outcomes. Pancreas.

[38] SpyGlass™ DS—Direct Visualization System. https://www.bostonscientific.com/content/gwc/en-US/products/direct-visualization-systems/spyglass-ds-direct-visualization-system.html.

[39] Wong J.C., Tang R.S., Teoh A.Y., Sung J.J., Lau J.Y. (2017). Efficacy and safety of novel digital single-operator peroral cholangioscopy-guided laser lithotripsy for complicated biliary stones. Endosc. Int. Open.

[40] Brewer Gutierrez O.I., Bekkali N.L.H., Raijman I., Sturgess R., Sejpal D.V., Aridi H.D., Sherman S., Shah R.J., Kwon R.S., Buxbaum J.L. (2018). Efficacy and Safety of Digital Single-Operator Cholangioscopy for Difficult Biliary Stones. Clin. Gastroenterol. Hepatol..

[41] Hendren S., Fiscella K. (2013). Patient Navigation Improves the Care Experience for Patients With Newly Diagnosed Cancer. J. Clin. Oncol..

[42] Harding M. (2015). Effect of nurse navigation on patient care satisfaction and distress associated with breast biopsy. Clin. J. Oncol. Nurs..

[43] Campbell C., Craig J., Eggert J., Bailey-Dorton C. (2010). Implementing and measuring the impact of patient navigation at a comprehensive community cancer center. Oncol. Nurs. Forum.

[44] Wells K.J., Battaglia T.A., Dudley D.J., Garcia R., Greene A., Calhoun E., Mandelblatt J.S., Paskett E.D., Raich P.C., Patient Navigation Research P. (2008). Patient navigation: State of the art or is it science?. Cancer.

[45] Schafer J.M., Swisher J. (2006). Clinical Intelligence: Cancer Care Coordination with Nurse Navigators.

[46] Gillis C., Wischmeyer P.E. (2019). Pre-operative nutrition and the elective surgical patient: Why, how and what?. Anaesthesia.

[47] Weggelaar-Jansen A., Broekharst D.S.E., de Bruijne M. (2018). Developing a hospital-wide quality and safety dashboard: A qualitative research study. BMJ Qual. Saf..

[48] Dowding D., Randell R., Gardner P., Fitzpatrick G., Dykes P., Favela J., Hamer S., Whitewood-Moores Z., Hardiker N., Borycki E. (2015). Dashboards for improving patient care: Review of the literature. Int. J. Med. Inform..

[49] Ivers N.M., Barrett J. (2018). Using report cards and dashboards to drive quality improvement: Lessons learnt and lessons still to learn. BMJ Qual. Saf..

[50] Chee T.T., Ryan A.M., Wasfy J.H., Borden W.B. (2016). Current State of Value-Based Purchasing Programs. Circulation.

[51] Porter M.E. (2006). Redefining Health Care: Creating Value-Based Competition on Results.

[52] Wong J., Chan I. Engaging Physicians Using Value Management Tools—NUHS Experience. Proceedings of the Institute for Healthcare Improvement National Forum.

[53] Lee V.S., Kawamoto K., Hess R., Park C., Young J., Hunter C., Johnson S., Gulbransen S., Pelt C.E., Horton D.J. (2016). Implementation of a Value-Driven Outcomes Program to Identify High Variability in Clinical Costs and Outcomes and Association With Reduced Cost and Improved Quality. JAMA.

[54] Shah J., Nwogu C., Vivian E., John E.S., Kedia P., Sellers B., Cler L., Acharya P., Tarnasky P. (2021). The Value of Managing Acute Pancreatitis With Standardized Order Sets to Achieve “Perfect Care”. Pancreas.

[55] Steele S., Branstetter H., Shah J., Acharya P., Avila N., Khan H., Reddy R., Muller M., Kedia P., Tarnasky P. Initial Imaging in Patients with Acute Pancreatitis: Impact of Quality Improvement. Proceedings of the World Congress of Gastroenterology at ACG.

[56] Benefits of Joint Commission Certification. https://www.jointcommission.org/benefits_of_joint_commission_certification/.

[57] Facts about The Joint Commission. https://www.jointcommission.org/facts_about_the_joint_commission/.

[58] DNV GL Healthcare Program Certifications. https://www.dnvgl.us/assurance/healthcare/certifications.html.

[59] Methodist Dallas Medical Center Named General Surgery Epicenter Focusing on Liver and Pancreas Robotic-Assisted Surgery. https://www.methodisthealthsystem.org/news-center/2015/february/methodist-dallas-medical-center-named-general-su/.

[60] Managing New Products: The Product Life Cycle. https://open.lib.umn.edu/principlesmarketing/chapter/7-2-managing-new-products-the-product-life-cycle/.

[61] Ethics: Advertising & Publicity. https://www.ama-assn.org/delivering-care/ethics/advertising-publicity.

